# SMILE and Wavefront-Guided LASIK Out-Compete Other Refractive Surgeries in Ameliorating the Induction of High-Order Aberrations in Anterior Corneal Surface

**DOI:** 10.1155/2016/8702162

**Published:** 2016-10-13

**Authors:** Min-jie Ye, Cai-yuan Liu, Rong-feng Liao, Zheng-yu Gu, Bing-ying Zhao, Yi Liao

**Affiliations:** ^1^Department of Ophthalmology, The First Affiliated Hospital of Anhui Medical University, Hefei, Anhui, China; ^2^Department of Ophthalmology, The Hospital of University of Science and Technology of China, Hefei, Anhui, China; ^3^Eye Institute of Xiamen University, Medical College of Xiamen University, Fujian Provincial Key Laboratory of Ophthalmology and Visual Science, Xiamen, Fujian, China

## Abstract

*Purpose*. To compare the change of anterior corneal higher-order aberrations (HOAs) after laser in situ keratomileusis (LASIK), wavefront-guided LASIK with iris registration (WF-LASIK), femtosecond laser-assisted laser in situ keratomileusis (FS-LASIK), and small incision lenticule extraction (SMILE).* Methods*. In a prospective study, 82 eyes underwent LASIK, 119 eyes underwent WF-LASIK, 88 eyes underwent FS-LASIK, and 170 eyes underwent SMILE surgery. HOAs were measured with Pentacam device preoperatively and 6 months after surgery. The aberrations were described as Zernike polynomials, and analysis focused on total HOAs, spherical aberration (SA), horizontal coma, and vertical coma over 6 mm diameter central corneal zone.* Results*. Six months postoperatively, all procedures result in increase of anterior corneal total HOAs and SA. There were no significant differences in the induced HOAs between LASIK and FS-LASIK, while SMILE induced fewer total HOAs and SA compared with LASIK and FS-LASIK. Similarly, WF-LASIK also induced less total HOAs than LASIK and FS-LASIK, but only fewer SA than FS-LASIK (*P* < 0.05). No significant difference could be detected in the induced total HOAs and SA between SMILE and WF-LASIK, whereas SMILE induced more horizontal coma and vertical coma compared with WF-LASIK (*P* < 0.05).* Conclusion*. FS-LASIK and LASIK induced comparable anterior corneal HOAs. Compared to LASIK and FS-LASIK, both SMILE and WF-LASIK showed advantages in inducing less total HOAs. In addition, SMILE also possesses better ability to reduce the induction of SA in comparison with LASIK and FS-LASIK. However, SMILE induced more horizontal coma and vertical coma compared with WF-LASIK, indicating that the centration of SMILE procedure is probably less precise than WF-LASIK.

## 1. Introduction

Laser in situ keratomileusis (LASIK) was first introduced by Pallikaris et al. in 1990 and has become presently the most common and effective refractive surgery to correct myopia, in which a stromal flap is created with a mechanical microkeratome, folded back, and repositioned. The main surgical step of this procedure is the flap creation [1], which induces corneal higher-order aberrations (HOAs) and may compromise postoperative visual quality [2]. In 1994, Liang et al. used a Shack-Hartmann wavefront sensor to describe HOAs in human eye and detect HOAs 3 years later [3, 4]. In 1999, wavefront-guided laser technology was introduced into the field of refractive surgery. Theoretically, this improvement allows an optimized correction not only of spherocylindrical errors but also of HOAs [5–7]. However, the induction of corneal HOAs has also been reported [1, 8, 9].

More recently, femtosecond laser is adopted to perform refractive surgery with high accuracy. Femtosecond laser-assisted laser in situ keratomileusis (FS-LASIK) and small incision lenticule extraction (SMILE) are new refractive procedures performed on the basis of femtosecond laser. During FS-LASIK procedure, femtosecond laser was used to create corneal flaps [5, 6, 10]. Its main advantage over mechanical microkeratomes is that femtosecond laser allows surgeons to customize the parameters of corneal flap, such as diameter, thickness, and hinge position, which may reduce the incidence of intraoperative complications, including irregular or buttonholed flaps and epithelial defects [7, 11, 12]. The femtosecond laser-created flaps also showed stronger adhesion at the interface and flap edge than microkeratome flaps [1, 13]. During the SMILE procedure, an intrastromal lenticule is created with the femtosecond laser and then removed through a small incision to avoid the creation of the flap. Therefore, this flapless and small incision extracted procedure may reduce the flap-related complications and sever fewer corneal nerves. Theoretically, SMILE should be a minimal invasive corneal refractive surgery [14, 15].

The total aberrations in eyes include corneal aberrations and internal aberrations, between which cornea contributes a critical part of HOAs. Although the change of internal aberrations could compensate the effect of induced corneal HOAs following refractive surgery [16], the anterior corneal surface is immediately influenced by refractive surgery and plays a dominant role in determining total HOAs [13, 15, 17, 18]. Anterior corneal surface is immediately influenced by refractive surgery. Consequently, the change of anterior corneal HOAs is suggested to be used as a way to evaluate optical quality after refractive surgery. Despite the fact that new technologies and minimal invasive approaches have been broadly applied in refractive surgery, the changes of anterior corneal HOAs after refractive surgeries have not been completely acknowledged. The aim of this study was to compare the changes of HOAs in anterior corneal surface among LASIK, WF-LASIK, FS-LASIK, and SMILE postoperatively. To our knowledge, this is the first study to compare anterior corneal HOAs among these four surgical procedures.

## 2. Patients and Methods

### 2.1. Patients

This prospective study comprised patients who were scheduled for LASIK from September 2012 to December 2013 at the hospital of the University of Science and Technology of China. All the patients were selected according to inclusion criteria, and 459 eyes of 230 myopic patients were enrolled, in which 82 eyes of 41 myopia patients, 119 eyes of 60 myopia patients, 88 eyes of 44 myopia patients, and 170 eyes of 85 myopia patients were enrolled in LASIK, WF-LASIK, FS-LASIK, and SMILE, respectively. The standard inclusion criteria for four procedures were set as follows: only eyes with myopic or myopic astigmatism errors less than −10.0 D spherical equivalent (SE) were included. Other inclusion criteria included at least 18 years of age, a stable refraction history for 1 year (the increase of the refractive error less than 0.5 D), and spectacle-corrected visual acuity of 20/20 or better. Exclusion criteria included previous refractive surgery, corneal disease, cataract, glaucoma, amblyopia, or retinal disease, diabetes mellitus, and connective tissue disease. This study was reviewed and approved by the Institutional Review Board at the First Affiliated Hospital of Anhui Medical University. All patients were informed of the details and risks of the procedures, and each patient provided informed consent in accordance with the World Medical Association Declaration of Helsinki.

The anterior corneal HOAs were measured by the Oculus Pentacam HR device (Oculus Inc.) preoperatively and 6 months after surgery, over the 6.0 mm diameter central corneal zone. Then, the aberrations of anterior corneal surface were calculated using the Zernike polynomials with an expansion up to the 6th order by the software of Pentacam system [19]. The values of HOAs were presented as root-mean-square (RMS, in micrometers). Each Zernike Coefficient used for statistical analysis was the average of at least five measurements. All examinations were performed by an experienced ophthalmic technician.

### 2.2. LASIK and WF-LASIK Procedures

The procedures were performed under topical anesthesia with two drops of proparacaine hydrochloride 0.5% (Alcaine) over a 5-minute interval. A rigid eyelid speculum was used to keep the eye open. The automated microkeratome (M2, Moria, France) was used to create a 110 *μ*m thickness hinged corneal flap. The flap was lifted with a spatula, and the stromal bed was exposed. Laser ablation was performed using the VISX S4 excimer laser system (Visx Inc., Santa Clara, CA, USA). The laser was fired on a dried corneal surface with the following operative parameters: energy fluency 160 mJ/cm^2^, emission wavelength 193 nm, and repetition rate 10 Hz. The optical zone was 6.00 mm and transition zone was 8.00 mm. All procedures were assisted by an eye tracker. Then, the flap was repositioned with a spatula. Afterward, irrigation was done under the flap with balanced salt solution. In WF-LASIK group, all HOAs were measured by Wavescan (Visx Inc.). Data from Wavescan were translated to the excimer laser system by a floppy disk.

### 2.3. FS-LASIK

All FS-LASIK procedures were performed by the VisuMax femtosecond laser system (Carl Zeiss Meditec AG, Germany) with a repetition rate of 500 kHz and a pulse energy of 130 nJ. The pulses were focused at a precise depth in the corneal tissue, and the laser pulses created microphotodisruption or a series of bubbles of water and carbon dioxide gas that in turn cleaved the tissue and created a plane of separation. The track distance and spot separation were 3.0 *μ*m during flap creation and 1.5 *μ*m during flap side-cutting. Side-cut angle and hinge angle were 90 degrees and 50 degrees, respectively. The flap diameter and thickness were 8.0 mm and 105 *μ*m, respectively. Once the creation of flap was completed, the excimer ablation of the stromal bed and the reposition of the flap were in a similar fashion as in routine LASIK procedure.

### 2.4. SMILE Procedure

In SMILE group, the femtosecond laser pulse energy, pulse repetition rate, the track distance, and spot separation were the same as for the FS-LASIK group. Four subsequent femtosecond incisions are performed in SMILE as follows: (1) the posterior surface of the refractive lenticule, (2) the vertical edge of the refractive lenticule, (3) the anterior surface of the refractive lenticule, and (4) a single small 90-degree angled side-cut incision with a circumferential length of 4.0 to 5.0 mm in the 12 o'clock position. In all cases, the depth of anterior surface of the lenticule was 120–130 *μ*m. For all myopic corrections, the optical zone size was 6.0 mm. The exact surgical maneuver was described elsewhere [15, 20].

### 2.5. Statistical Analysis

Paired Student's *t*-tests were used to compare preoperative and postoperative anterior corneal HOAs. One-way analysis of variance (ANOVA) was used for comparisons across the groups. Changes in the aberrations were calculated as the differences between postoperative and baseline values. *P* value of less than 0.05 was considered statistically significant. The statistical power was calculated for sample size of each group in the PASS 11.0 software, and the statistical powers of all sample sizes were over 70.0%. The value of significance was set as 0.05.

## 3. Results

There were no statistically significant differences between the LASIK, WF- LASIK, FS-LASIK, and SMILE groups in the mean preoperative spherical equivalent (−4.91 ± 1.81, −4.82 ± 1.55, −5.43 ± 2.32, and −5.03 ± 1.89, resp.) and mean age (25.12 ± 4.70, 24.22 ± 3.69, 24.16 ± 4.48, 25.25 ± 4.20, resp.) at the time of treatment (shown in Table 1).

Table 2 showed anterior corneal HOAs over 6 mm diameter of central corneal zone preoperatively and at 6 months after surgery for each of the four groups. The HOAs were measured by the Oculus Pentacam device. It has been reported that Pentacam is a noninvasive anterior segment tomographer with high repeatability and reproducibility across comprehensive assessments [21, 22]. Preoperative HOAs were not statistically significantly different among these four groups, but postoperative HOAs were. The changes in HOAs induced by the surgeries were shown in Table 3. There was a significant (*P* < 0.001) increase in total HOAs 6 months postoperatively in all groups (LASIK: 0.11 ± 0.09 *μ*m; WS-LASIK: 0.07 ± 0.06 *μ*m; FS-LASIK: 0.10 ± 0.60 *μ*m; SMILE: 0.07 ± 0.07 *μ*m), as well as in SA (LASIK: 0.02 ± 0.35 *μ*m; WS-LASIK: 0.19 ± 0.20 *μ*m; FS-LASIK: 0.29 ± 0.21 *μ*m; SMILE: 0.16 ± 0.16 *μ*m). Significant difference was also found in horizontal coma 6 months postoperatively (LASIK: 0.02 ± 0.35 *μ*m; WS-LASIK: −0.06 ± 0.22 *μ*m; FS-LASIK: 0.02 ± 0.49 *μ*m; SMILE: 0.08 ± 0.18 *μ*m). For the induced vertical coma, it was also significantly different among all groups (LASIK: −0.10 ± 0.32 *μ*m; WS-LASIK: −0.02 ± 0.36 *μ*m; FS-LASIK: −0.18 ± 0.54 *μ*m; SMILE: −0.22 ± 0.22 *μ*m).

**Figure 1:**
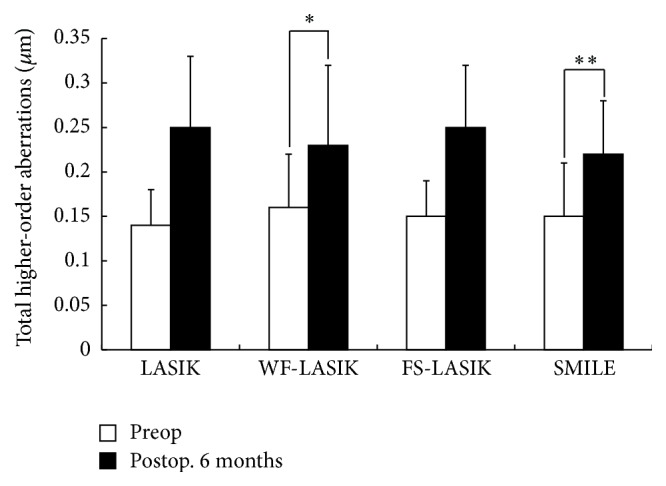
Comparison of the total higher-order aberrations (HOAs) changes in four groups. Comparison of the induced total HOAs between wavefront-guided LASIK and both LASIK and femtosecond laser-assisted LASIK (^*∗*^ *P* values are 0.001 and 0.023, resp.). Comparison of the induced total HOAs between small incision lenticule extraction (SMILE) and both LASIK and femtosecond laser-assisted LASIK (^*∗∗*^ *P* values are < 0.001 and 0.018, resp.).

**Figure 2:**
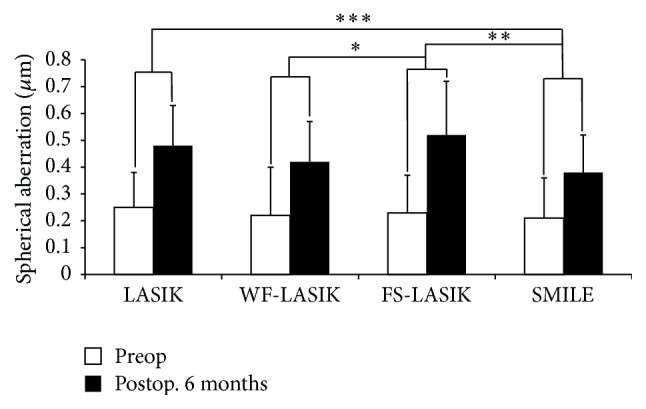
Comparison of the spherical aberration (SA) changes in four groups. Comparison of the induced SA between wavefront-guided LASIK and femtosecond laser-assisted LASIK (^*∗*^ *P* value is 0.001). Comparison of the induced SA between small incision lenticule extraction and both femtosecond laser-assisted LASIK (^*∗∗*^ *P* value is < 0.001) and LASIK (^*∗∗∗*^ *P* value is 0.030).

**Figure 3:**
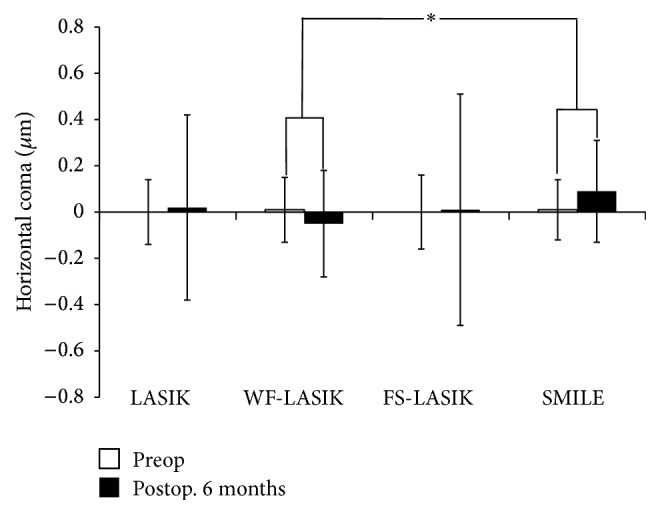
Comparison of the horizontal coma changes in four groups. Comparison of the induced horizontal coma between small incision lenticule extraction and wavefront-guided LASIK (^*∗*^ *P* value is 0.001). Positive and negative values indicate an average positive or negative shift, respectively, in horizontal coma relative to preoperative values.

**Figure 4:**
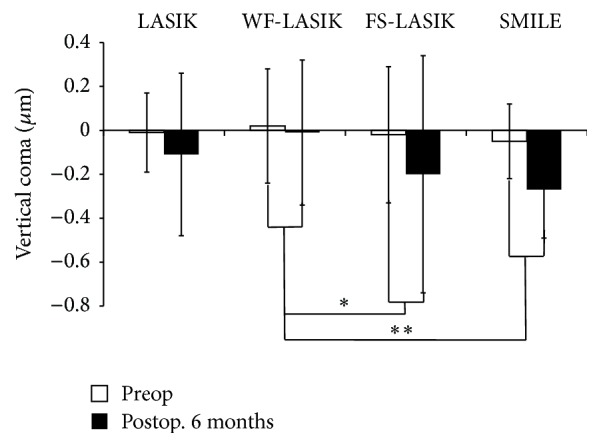
Comparison of the vertical coma changes in four groups. Comparison of the induced vertical coma between wavefront-guided LASIK and both femtosecond laser-assisted LASIK (^*∗*^ *P* value is 0.015) and small incision lenticule extraction (^*∗∗*^ *P* value is < 0.001). Positive and negative values indicate an average positive or negative shift, respectively, in vertical coma relative to preoperative values.

One-Way ANOVA test showed significant discrepancies in the induced anterior corneal HOAs among four groups 6 months postoperatively. In addition, multiple comparison procedures (Bonferroni *t*-test) were used to compare changes in HOAs among the groups. SMILE induced less total HOAs and SA compared with LASIK and FS-LASIK (*P* values were < 0.001 and 0.018, resp.). This was similar in the induced total HOAs or SA between SMILE and WF-LASIK (Figures 1 and 2). However, significant differences were found in the induced horizontal coma and vertical coma between SMILE and WF-LASIK (*P* values were 0.001 and <0.001, resp.; Figures 3 and 4). WF-LASIK induced less total HOAs compared with LASIK (*P* = 0.023, Figure 1) and fewer SA compared with FS-LASIK (*P* = 0.001, Figure 2). In addition, we also found significantly different changes between WF-LASIK and FS-LASIK in terms of vertical coma (*P* = 0.015, Figure 4). Between LASIK and FS-LASIK, induced total HOAs, SA, horizontal coma, and vertical coma were all comparable.

## 4. Discussion

Laser corneal refractive surgery has become a safe and reliable option for the correction of myopia. However, the induction of significant amounts of HOAs has been described as an important side effect of this surgical option [23–25]. This aberrometric phenomenon has been associated with several factors, such as corneal biomechanical changes, the use of inappropriate ablation algorithms, flap formation, and decentration of the ablation [26–28].

In this present study, our results illustrated that the induced HOAs were significantly different among four treatment groups in favor of SMILE and WF-LASIK groups. Both SMILE and WF-LASIK induced less total HOAs and SA compared with LASIK and FS-LASIK, respectively, yet with no significant difference found in the change of SA between WF-LASIK and LASIK. There was no difference in the induced total HOAs or SA between SMILE and WF-LASIK, though SMILE induced more horizontal coma and vertical coma compared with WF-LASIK. Why did SMILE induce more horizontal and vertical coma than WF-LASIK? The authors assumed that there were several possible explanations for this result: (1) WF-LASIK was designed to compensate the induction of HOAs, with its advantage in reducing coma induction in comparison with other refractive surgeries without wavefront-guided laser technology. (2) SMILE does not include the iris registration technology that compensates for pupillary cyclotorsion and offset to ensure good centration. Furthermore, the centration of the SMILE procedure is more likely to be less precise than the automated centration operated by the eye tracker in an excimer laser [29]. (3) it is known that coma aberrations represent the characteristics of the asymmetry of eyes and meanwhile reflect the irregular, tilt, and decentration [30]. In SMILE, the astigmatism corrections result in an oval posterior surface of the lenticule. Therefore, the diameter of cleavage plane in the steep axis was smaller than in the flat axis [15]. Extraction of the oval lenticule from the cornea may be one of the important sources of asymmetry in SMILE surgery. Moreover, compared to 2.0 mm transition zone around the optical zone in WF-LASIK, the vertical edge of the refractive lenticule in SMILE may also induce the postoperative coma. We found that there was no difference in the induced total HOAs or SA between SMILE and WF-LASIK. The advantage in the lesser induction of coma aberrations of WF-LASIK may be offset by the advantages in lesser corneal biomechanical responses and corneal wound-healing responses of SMILE. Thus, the authors assumed that SMILE is essentially equivalent to WF-LASIK in anterior corneal total HOAs induction, although there was difference of coma induction.

In contrast to LASIK flaps created with mechanical microkeratomes, multiple studies have suggested that the geometrically planar configuration of bladeless flaps created by femtosecond laser confers advantages over microkeratome flaps, including the induction of fewer HOAs [31, 32]. Conversely, our data showed that there was no significant difference in the induced anterior corneal HOAs between LASIK and FS-LASIK. This is consistent with Calvo et al. [33], who detected no difference in corneal total HOAs, coma, SA, or trefoil between flaps created with Hansatome microkeratome and femtosecond laser at any time point during three years after LASIK. Likewise, Alió and Piñero found no statistically significant differences in spherical-like or coma-like root-mean-square corneal aberrometry among the Moria M2, Carriazo-Pendular microkeratomes, and IntraLase femtosecond laser 3 months after LASIK [34]. Similarly, Muñoz et al. found increases in anterior corneal aberrations between femtosecond laser and Carriazo-Barraquer microkeratome after LASIK in 98 eyes [7]. Chan et al. found that the femtosecond laser group had fewer SA and coma aberrations and more trefoil aberrations than the Hansatome microkeratome group, whereas these differences were statistically significant at 3 months but not at 6 or 12 months after LASIK [35]. Several explanations for the result we obtained regarding the comparable induction of higher-order aberrations between LASIK and FS-LASIK were proposed: (1) Porter et al. [36] concluded that the majority of SA induced by LASIK was primarily caused by laser ablation rather than microkeratome incision. Consistently, this conclusion was also supported by Oshika and coworkers [37]. This suggests that the induction of anterior corneal HOAs after surgery may not be related to the method of flap creation. (2) Although there was no statistically significant difference, the preoperative SE of FS-LASIK (−5.43 ± 2.32) is slightly higher than that of LASIK (−4.91 ± 1.81), which may induce more corneal biochemical alterations, wound-healing responses, and aberrations after surgery and thus reduce the advantage of femtosecond laser-created flap. (3) Just as mentioned above, flaps created by femtosecond laser confer advantages over microkeratome flaps. However, compared with LASIK, these advantages of FS-LASIK may subside and disappear in 6 months.

Compared to FS-LASIK, SMILE is an all-in-one femtosecond procedure, during which no excimer laser is needed to correct the refractive errors. Although a flap is created, it is not cut completely or lifted which helps the epithelium at the incision edge heal relatively more quickly. The small incision may induce less corneal wound-healing response, which was thought to be a major factor contributing to a number of the complications of corneal refractive surgery, including the induction of HOAs. Furthermore, the femtosecond laser is less influenced by peripheral energy loss as it creates the refractive lenticule as likely occurs with an excimer laser. These advantages may result in less induction of total HOAs and SA in SMILE over FS-LASIK in our data.

In conclusion, all procedures lead to increase in anterior corneal total HOAs and SA 6 months postoperatively. FS-LASIK and LASIK induced comparable anterior corneal HOAs. Compared to LASIK and FS-LASIK, both SMILE and WF-LASIK showed advantages to induce significantly less total HOAs. In terms of SA, though a trend of reduction was observed in both SMILE and WF-LASIK, significant changes can only be detected in the comparison between SMILE and LASIK/FS-LASIK. However, the significant discrepancy between SMILE and WF-LASIK in horizontal coma and vertical coma indicates that the centration of SMILE procedure is probably less precise than WF-LASIK.

## Figures and Tables

**Table 1:** Preoperative patient characteristics (mean ± SD).

Parameter	LASIK	WF-LASIK	FS-LASIK	SMILE	*P* value
Age (Y)	25.12 ± 4.70	24.22 ± 3.69	24.16 ± 4.48	25.25 ± 4.20	0.088
SE (D)	−4.91 ± 1.81	−4.82 ± 1.55	−5.43 ± 2.32	−5.03 ± 1.89	0.125
HOAs (*μ*m)					
Total HOAs	0.14 ± 0.04	0.16 ± 0.06	0.15 ± 0.04	0.15 ± 0.06	0.084
SA	0.25 ± 0.13	0.22 ± 0.18	0.23 ± 0.14	0.21 ± 0.15	0.441
Horizontal coma	0.00 ± 0.14	0.01 ± 0.14	0.00 ± 0.16	0.01 ± 0.13	0.839
Vertical coma	−0.01 ± 0.18	0.02 ± 0.26	−0.02 ± 0.31	−0.05 ± 0.17	0.095

LASIK: laser in situ keratomileusis; WF-LASIK: wavefront-guided laser in situ keratomileusis with iris registration; FS-LASIK: femtosecond laser-assisted laser in situ keratomileusis; SMILE: small incision lenticule extraction; HOAs: higher-order aberrations; SA: spherical aberration.

**Table 2:** Preoperative and 6-month postoperative anterior corneal HOAs with 6 mm diameter central corneal zone (mean ± SD, *μ*m).

Procedure	Total HOAs	SA	Horizontal coma	Vertical coma
*LASIK*				
Preoperative	0.14 ± 0.04	0.25 ± 0.13	0.00 ± 0.14	−0.01 ± 0.18
6-month postoperative	0.25 ± 0.08	0.48 ± 0.15	0.02 ± 0.42	−0.11 ± 0.37
*P* value	<0.001	<0.001	0.666	0.007
*WF-LASIK*				
Preoperative	0.16 ± 0.06	0.22 ± 0.18	0.01 ± 0.14	0.02 ± 0.26
6-month postoperative	0.23 ± 0.09	0.42 ± 0.15	−0.05 ± 0.23	−0.01 ± 0.33
*P* value	<0.001	<0.001	0.004	0.458
*FS-LASIK*				
Preoperative	0.15 ± 0.04	0.23 ± 0.14	0.00 ± 0.16	−0.02 ± 0.31
6-month postoperative	0.25 ± 0.07	0.52 ± 0.20	0.01 ± 0.57	−0.20 ± 0.54
*P* value	<0.001	<0.001	0.754	0.003
*SMILE*				
Preoperative	0.15 ± 0.06	0.21 ± 0.15	0.01 ± 0.13	−0.05 ± 0.17
6-month postoperative	0.22 ± 0.06	0.38 ± 0.14	0.09 ± 0.22	−0.27 ± 0.22
*P* value	<0.001	<0.001	<0.001	<0.001

Paired *t*-tests were used to compare pre- and postoperative aberrations. LASIK: laser in situ keratomileusis; WF-LASIK: wavefront-guided laser in situ keratomileusis with iris registration; FS-LASIK: femtosecond laser-assisted laser in situ keratomileusis; SMILE: small incision lenticule extraction; HOAs: higher-order aberrations; SA: spherical aberration.

**Table 3:** The changes in anterior corneal HOAs with 6 mm diameter central corneal zone (mean ± SD, *μ*m).

Change	LASIK	WF-LASIK	FS-LASIK	SMILE	*P* value
Total HOAs	0.11 ± 0.09	0.07 ± 0.06	0.10 ± 0.60	0.07 ± 0.07	<0.001
SA	0.23 ± 0.17	0.19 ± 0.20	0.29 ± 0.21	0.16 ± 0.16	<0.001
Horizontal coma	0.02 ± 0.35	−0.06 ± 0.22	0.02 ± 0.49	0.08 ± 0.18	0.002
Vertical coma	−0.10 ± 0.32	−0.02 ± 0.36	−0.18 ± 0.54	−0.22 ± 0.22	<0.001

One-Way ANOVA test was used for comparisons across the groups. LASIK: laser in situ keratomileusis; WF-LASIK: wavefront-guided laser in situ keratomileusis with iris registration; FS-LASIK: femtosecond laser-assisted laser in situ keratomileusis; SMILE: small incision lenticule extraction; HOAs: higher-order aberrations; SA: spherical aberration.
